# 3D Printed Prostheses: The Path from Hype to Reality

**DOI:** 10.33137/cpoj.v6i2.42141

**Published:** 2023-12-22

**Authors:** J Erenstone

**Affiliations:** Mountain Orthotic and Prosthetics Services, Lake Placid, NY USA.

**Keywords:** Orthotics, Prosthetics, 3D Printing, Additive Manufacturing, Digital Workflow, Rehabilitation

## Abstract

3D printing has an over forty-year history but has only become popular in the last fifteen years with the expiration of restrictive patents which allowed open access and unfettered innovation by a broad range of technology developers. During the last decade interest in prostheses made by 3D printing has grown in popularity. The interest in devices has followed the Gartner Hype Cycle as 3D printing companies and associated organizations have used popular claims about 3D printed prostheses to increase their own company's popularity. These claims created unrealistic expectations which outran the early-stage limitations of the technology, causing disillusion. Currently, the industry is moving beyond these limitations and the field seems to be advancing at a sustainable rate. This article provides an understanding of the history of popular misconceptions surrounding the technology. It provides a basis for separating the myth from reality in 3D printing technology so the reader can question the popular preconceived ideas and find the real value. With a greater understanding of the past, one can apply lessons to present technology use and guide the direction of future 3D printing. This paper will also discuss lessons applicable to both high and low-income countries along with providing recommendations for the future development.

## INTRODUCTION

The field of prosthetics has undergone a remarkable transformation in recent years, with 3D printing technology emerging as an innovation that promises to revolutionize the design and production of prosthetic devices. This paper explores the history of the use of 3D printing and how popular perceptions influenced the development and application of the technology. The observations and perspectives in this paper are based on the author's first-hand experience integrating the technology into clinical practice, as well as experience founding multiple related organizations and companies.

An advisory firm named Gartner developed a graphical representation called the “Hype Cycle” which tracks the adoption and maturity of emerging technologies and concepts within industries like 3D printing, also known as adaptive manufacturing. The Hype Cycle consists of five phases: Innovation Trigger, Peak of Inflated Expectations, Trough of Disillusionment, Slope of Enlightenment, and the Plateau of Productivity.^1^ 3D printing and 3D printed prosthetics have followed the phases of the hype cycle and this framework provides a helpful guide to understand their recent histories. With an understanding of the perceptions and mis-perceptions that were created, the O&P field is in a better position to understand how to effectively use the technology in the future.

## HISTORY

Before discussing the hype cycle and how it relates to the popularity of 3D printed prostheses, we need to acknowledge that 3D printing was around long before it was popular. Technology used in printing can be traced to a 1951 patent which used projected light to harden a photo-sensitive colloid.^2^ Then, in 1981 Japanese inventor Hideo Kodama created a device that used ultraviolet lights to harden polymers to create solid objects. In 1984 Charles “Chuck” Hull altered the concept and used Kodama's UV lamps to cure photosensitive resin layer-by-layer to create a part. This technology was labeled stereolithography. From this additive technology other deposition processes were developed, including ones that are used today. These are broadly defined by ISO/ASTM 52900:2021 into seven categories: 1) Binder Jetting; 2) Directed Energy Deposition; 3) Material Extrusion; 4) Material Jetting; 5) Powder Bed Fusion; 6) Sheet Lamination; and (7) Vat Photopolymerization.^3^

In the field of prosthetics, 3D printing has been used for more than 30 years. In 1992, a researcher at Northwestern University named Josh Rolock developed a technology to fabricate sockets named SQUIRT-Shape as part of his PhD research.^4^ Rolock continued to develop this technology throughout the 1990s. This early additive technology allowed the one-step fabrication of prosthetic sockets and functions in a similar way to the most popular 3D printed socket fabrication used today.

## MASS POPULARITY

Even though the history of 3D printing spans several decades, its mass popularity didn't grow until 2009, when the now famous Stratasys' patent expired. Suddenly it became possible for a company to produce and sell simple material extrusion printers (commonly known as FDM printers) without infringement of Stratasys' intellectual property. Soon after this expiration, an open-source project called Reprap acted as a technology trigger when numerous people started to use the designs to develop their own low-cost printers.^5^ Some of the more functional designs were used to found companies which sold these low-cost printers. The expectations of this technology increased exponentially as people made objects with their printers and posted about them on social media for others to appreciate. Some companies, like Makerbot, launched large marketing campaigns which drew the attention of mass media. They started to compare the adoption of 3D printers to adoption of PC computers and promoted their goal of having a printer in every household.^6^

3D printed prosthesis entered the media streams in December 2012 with the publishing of the “The First 3D Printed Prosthetic Hand for a Child” made by Ivan Owen. His project was promoted in Makerbot's marketing material including the campaign for the opening of its second retail store. This “feel good” humanitarian story offered a counter story to the troubling media reports of 3D printed guns and became a popular human-interest story which was published broadly in the media and served as a triggering event for its popularity.^7^ During this time an online community named “e-NABLE”, which was founded by Jon Schull, promoted designs and messages that anyone with a low cost printer could download the designs, print prosthetic hands, and help children in need.^8^ In reality, the vast majority of devices were used only for a short time and have been found not satisfactory for a user's daily functional tasks and activities.^9^ Over the next couple of years, the popularity of 3D printed prostheses (in tandem with 3D printers in general) grew into the “peak of inflated expectation” on the hype cycle. This peak of public attention corresponded to high stock prices of companies like Stratasys^10^ and 3D Systems.^11^ Stratsys reached its all-time high on January 03, 2014 at a price of $136.46^12^ and 3D Systems peaked on the same day at $96.42.^13^ Afterward their stock prices dropped quickly and have yet to return in value.

My personal history with 3D printing started in 2010 by utilizing printed parts in designing adaptive sport orthotic and prosthetic devices. The technology was very advantageous in prototyping and fabricating small quantity sport components. Printing greatly improved fabrication time compared to conventional methods.

In September of 2014, a YouTube video was published by an e-NABLE member titled “A $50 3D-Printed Prosthesis Compared to a $42,000 Myoelectric Prosthesis.”^14^ This dramatic and inaccurate comparison measured the cost of the raw materials for a 3D printed hand to the full cost of a myoelectric arm billed to an insurance carrier in the price-regulated US healthcare system. It created a lot of hype in the media and enshrined the trope that “3D printed hands cost $50.” Tropes with similar dollar amounts are repeated to this day and cause misperception of the cost involved in prosthetic care. In response to the inaccuracies, I reached out to Schull with my concerns. The e-NABLE founder thanked me for reaching out, mentioned that no other prosthetist had contacted him yet, and asked me to post my thoughts on the e-NABLE google community which led to regular engagement within the community over the next couple years.

Through this time period, I worked to educate the well-meaning volunteers about complexities of prosthetic care while learning about 3D printing technology and its potential. The exchange of ideas was rewarding, but also frustrating to see that the tropes and misperceptions persisted due to the loose organizational structure of the online community. In this time period many 3D printing companies started to struggle through the “Trough of Disillusionment” as people realized that the low-cost 3D printers of that time were finicky to use and regularly produced inferior products when compared to conventional fabrication methods. Everyone agreed that tremendous potential existed in 3D printing, but it seemed the technology was too early in its development to be viable for most applications.

Through engagement with these companies, I was able to identify some applications in the prosthetic field which were advantageous for my own clinical practice, including designing and fabricating diagnostic sockets, flexible inner sockets, and cosmetic covers. The successes found in the clinic inspired the founding of a 3D printing company named Create O&P^15^ which has since been acquired by PVA Med. The company was in step with the rest of the 3D printing field at that time which seemed to be on the “Slope of Enlightenment.” For this technology to be adopted and effective, it needed to be supplied as a complete end to end solution which included scanning, CAD software, printer, application knowledge, and support. A complete system, based around the creation of prosthetic devices with a proven clinical application, the prosthetic field was willing to embrace the technology and incorporate it into clinical care. While this comprehensive approach did not scale quickly, in the right hands, over time, it started to meet some of the expectations that were promised at the peak of the hype.

3D printing is a popular term while Additive Manufacturing (AM) is a more technological term used by established researchers, engineers and industrialists. AM technology is not likely to allow anyone with a 3D printer to provide “amazingly low-cost prosthetic care” that was promised. The need for trained clinicians remains. However, now that this technology is in the hands of professionals who understand the complexity and challenges of clinical prosthetic care, the technology is on the slow and steady path to productivity and will have a firm place in the future of O&P care. Every O&P provider should be introduced to the technology and have a basic understanding of how-to 3D scan and digitally design devices in CAD. This foundation of knowledge will help them incorporate the technology into their daily practice. At this point, there are numerous people in the O&P field who are utilizing AM regularly, but it is not yet the majority. Greater adoption requires companies to develop robust end to end workflows which provide a complete road map from initial patient encounter to the completion of a deliverable definitive device. The companies that provide this comprehensive workflow will have an enduring presence in the market and facilitate more regular use of AM by practitioners in their practices. This is the path that leads to popular use of the technology and achieves the “Plateau of Productivity,” as identified in the Hype Cycle.

## LESSONS LEARNED

A few years ago, I was in the mindset that I could completely give up modifying (rectifying) patient models made from plaster and only work in the digital space going forward. Being familiar with several types of CAD software and having experience with a wide range of AM, my typical workflow consisted of making prototypes (diagnostic devices) on low cost FDM printers in my own facility and then forwarding these designs to central fabrication (AM service bureaus) with expensive powder bed fusion printers for definitive devices. It was exciting to be done with plaster and never wash white dots off my shoes again. However, when discussing these plans with colleagues, it was pointed out that a dogmatic approach to only using digital technology was going to be less efficient and limiting in the care that could be provided.

Most O&P fabrication technologies have been used in the field for decades. Numerous tools and strategies have been developed around these methods and the bugs have already been worked out of the process. It doesn't make sense to give up on comfortable processes and ignore the wealth of knowledge and experience acquired over decades. These days, every time I start a project, I ask myself, which parts are best done using digital technology and which parts are better done using traditional methods. More often than not, a hybrid approach which uses a combination of digital technology and traditional methods is the right answer. For example, I regularly use scanning, CAD, and AM to make a thin-walled socket which prints quickly, then reinforce the outer surface of this socket with a conventional carbon fiber or fiberglass lamination to add strength and conventional alignment componentry.

This mindset has carried over to my work in low-income countries and my work with an organization named Operation Namaste.^16^ The organization has done most of its work in Nepal where there are several well outfitted prosthetic fabrication facilities for patients who have means to travel. In these cases, there is no need to introduce new digital technology that is still in early stage development and not fully vetted.

Meanwhile, there are people in Nepal who still struggle to travel to urban areas with established clinics. For these cases Operation Namaste is developing a compact mobile lab which utilizes digital technology. A computer and 3D printer are much smaller and easier to transport than a plaster lab, an oven and vacuum forming system. Our mobile lab, which we call “Limbkit,” includes all the equipment needed to fabricate transtibial prosthesis and can be packaged into a case that is transportable by SUV. In this system we are using a low-cost FDM 3D printer to make clear thinned-wall diagnostic sockets out of PETG, fitting them as a diagnostic socket to confirm the fit, then adding a prescribed amount of fiberglass reinforcement to strengthen them into definitive devices. The kit doesn't require a plaster lab because Operation Namaste has developed its own web-based CAD software named “Collaborative CAD”. In the future, when this system is fully developed it will allow prosthetists to fit devices in numerous low resource settings and conflict zones where care wasn't previously available. We are not trying to disrupt established functional clinical care, but instead, look to extend care into areas with unmet needs. Any time a new technology can allow practitioners to provide care where they have not been able to previously, the technology is much more likely to be adopted. Operation Namaste is working to extend current care into geographical regions where care was previously lacking. Meanwhile other AM developers are forging the pathway into new types of clinical care elsewhere where the technology was not previously available.

## CALL TO ACTION

For Additive Manufacturing to reach its potential in the field of prosthetics, AM developers need to keep in mind the temperament of the practitioners using the technology. They need to develop more end-to-end workflows which fabricate orthotic and prosthetic devices that are difficult or impossible to make by traditional fabrication methods. When practitioners see that they will be able to reliably provide better care, the majority will embrace new digital technologies and be willing to shift away from the traditional methods that they are currently comfortable with.

Additionally, when it comes to designing and fabricating structural components there are engineering limitations that need to be overcome or properly taken into account. There needs to be an increase in consistent Isotropic bonding of material in AM devices so the properties are consistent with the properties found in other manufacturing processes such as vacuum forming or CNC milling. This is especially a concern in FDM printed sockets where the chopped layer in the vertical (z direction) and rapid cooling glass transition level between the layers makes the z direction significantly weaker than the x and y directions and causes reduced strength of the part in this orientation.^17^ The lack of consistent bonding in all directions makes designing new innovative load bearing components difficult because the material dynamics are not predictable and hard to model. Powder bed fusion printers (more commonly known as SLS or MJF printers) use methods that are more isotopic than FDM, but improvements are needed in fabrication consistency and part-to-part quality control with these processes as well.

Furthermore, regarding prosthetic sockets, there is a substantial knowledge gap surrounding standardized methods for the mechanical testing. This absence of standardized test methods means the structural properties of these sockets are not clearly defined or understood. This causes difficulty for new socket technology to be designed because designers don't know the strength requirements necessary in their design. In other words, even if AM parts become isotropic and consistent, the socket innovators do not have enough guidance to fully understand strength requirements to incorporate into their new designs. To begin addressing this dilemma, in 2020 a multidisciplinary group of professionals was assembled by the AOPA and has published a white paper called “Mechanical testing of transtibial prosthetic sockets: A discussion paper from the American Orthotic and Prosthetic Association Socket Guidance Workgroup.” This group aims to inspire researchers to narrow the gaps of knowledge required to make measurable standards for socket strength.^18^

With these types of improvements, additive manufacturing and other digital technology have the ability to achieve their potential and likely displace the current traditional methods. With a good understanding of the necessary requirements, AM can be on the path to achieving the improvement necessary to convince the majority of the practitioners in the field to use it routinely. In the meantime, it is clear that AM has a place in our field today and this place will grow every year. However, the field should not abandon the currently successful, traditional methods until AM progresses further. Instead, we should take a hybrid approach of using 3D printing (AM) for some aspects of fabrication and traditional methods for the rest.

## DECLARATION OF CONFLICTING INTERESTS

The author is the Founder of the Charity Operation Namaste https://www.operationnamaste.org/ and the Owner of Mountain Orthotic and Prosthetic Services in Lake Placid, USA.

## SOURCES OF SUPPORT

None.

## AUTHOR SCIENTIFIC BIOGRAPHY

**Figure:**
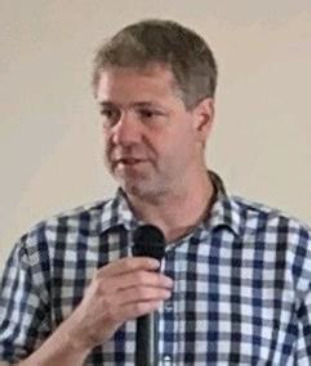


Jeffrey Erenstone has B.S. in Biology from The University of New Hampshire and two post-bachelor certificates in Prosthetic and Orthotic Practitioners from Newington and Century Colleges. He has been a certified prosthetist and orthotist (CPO) for nearly 20 years. He has owned his clinical practice named Mountain Orthotic and Prosthetics Services for 16 years which is in Northern New York. He serves as the chair of the American Orthotic and Prosthetic Association (AOPA) Digital O&P Committee and Socket Guidance Workgroup. He is the founder and president of the non-profit known as Operation Namaste. Jeff has devoted years to improving prosthetic care around the world, especially in LMICs. He is a well-known innovator of digital technology for prosthetic care and is working on systems of making silicone prosthetic liners in LMICs and a mobile prosthetic lab called the Limbkit.
